# Liquid Chromatography/Tandem Mass Spectrometry Method for Quantitation of Cremophor EL and Its Applications

**DOI:** 10.1155/2013/135613

**Published:** 2013-07-28

**Authors:** V. Vijaya Bhaskar, Anil Middha

**Affiliations:** Department of Pharmacy, Jagadishprasad Jhabermal Tibrewala University, Vidyanagari, Jhunjhunu, Rajasthan 333001, India

## Abstract

A rapid sensitive and selective MRM based method for the determination of Cremophor EL (CrEL) in rat plasma was developed using liquid chromatography/tandem mass spectrometry (LC-MS/MS). CrEL and polypropylene glycol (internal standard) were extracted from rat plasma with acetonitrile and analysed on C18 column (XBridge, 50 × 4.6 mm, 3.5 **μ**m). The most abundant molecular ions corresponding to PEG oligomers at *m/z* 828, 872, 916 and 960 with daughter ion at *m/z* 89 were selected for multiple reaction monitoring (MRM) in electrospray mode of ionisation. Plasma concentrations of CrEL were quantified after administration through oral and intravenous routes in male sprague dawley rats at a dose of 0.26 g/kg. The standard curve was linear (0.9972) over the concentration range of 1.00 to 200 **μ**g/mL. The lower limit of quantitation for CrEL was 1.00 **μ**g/mL using 50 **μ**L plasma. The coefficient of variation and relative error for inter and intra assay at three QC levels were 0.69 to 9.21 and −7.60 to 4.74 respectively. A novel proposal was conveyed to the scientific community, where formulation excipient can be analysed as qualifier in the analysis of NCEs to address the spiky plasma concentration profiles.

## 1. Introduction

**Figure 1:**
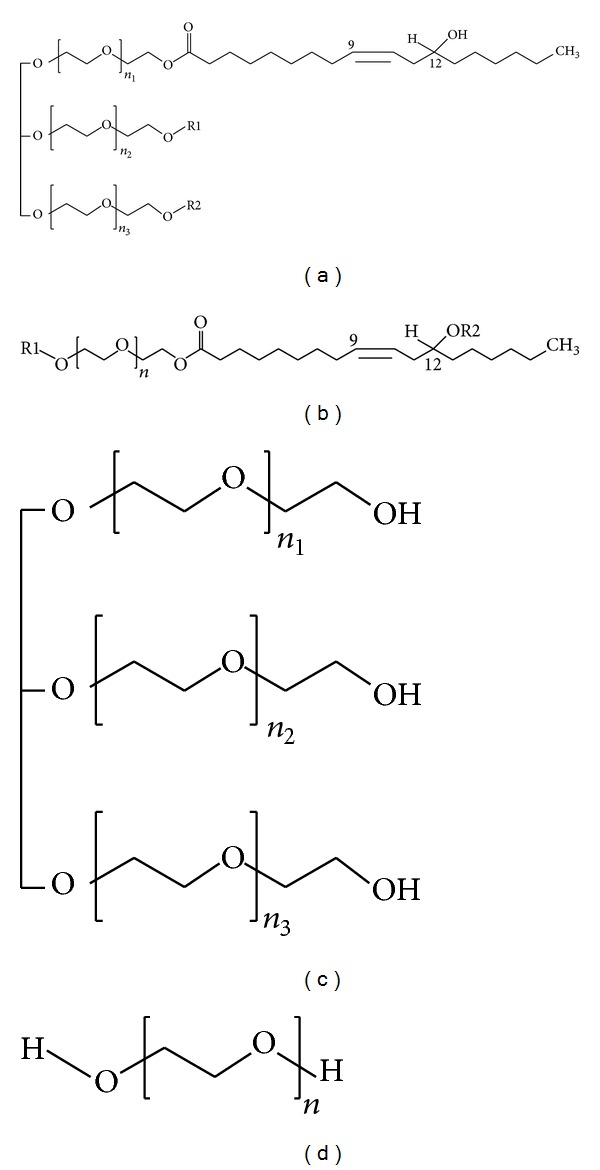
Components of CrEL according to [3]; hydrophobic portion consisting of (a) glycerol polyoxyethylene ricinoleate esters (R1, R2 corresponds to ricinoleic acid). (b) polyoxyethylene ricinoleate esters; hydrophilic portion consisting of (c) glycerol polyoxyethylene ether (d) free polyethyleneglycol.

In early stages of drug discovery, pharmacokinetic and drug metabolism and disposition studies were conducted in rodents, as they were relatively inexpensive and can be easily acquired and handled [1]. In a typical pharmacokinetic (PK) study, new chemical entities (NCEs) are administered to rats via intravenous and oral routes. Serial blood samples are collected and analysed by liquid chromatography/tandem mass spectrometry (LC-MS/MS). Few NCEs have spiky plasma concentration profiles and various reasons for such profiles could be due to enterohepatic circulation or discrepancies in sample collection/sample processing. Spiky profiles in elimination phase will lead to inaccurate quantification of PK parameters. Extensive studies needed to be carried to characterize enterohepatic circulation behavior of test compounds. Drugs that undergo enterohepatic cycling to a significant extent include colchicine, phenytoin, leflunomide, and tetracycline antibiotics. As formulation excipients have fixed plasma concentration profiles irrespective of NCEs dosed, monitoring the plasma concentration levels of excipient along with NCEs will help to take a decision on the spiky plasma concentration profiles of NCEs. A thoroughly developed and validated bioanalytical method is required to fix the plasma concentration profile and understand the pharmacokinetic disposition of formulation excipient studied. Integrity of results from pharmacokinetic studies can be cross-verified if formulation excipients that had fixed plasma concentration profile/PK parameters are monitored along with the test compound studied. Polyoxyethyleneglycerol triricinoleate (Cremophor EL: CrEL) is a liquid product formed by the reaction of ethylene oxide with castor oil at a molar ratio of 35 : 1 [2]. A major component (~80%) of CrEL consists of hydrophobic glycerol polyoxyethylene ricinoleate and polyoxyethylene ricinoleate (Figures 1(a) and 1(b)). The remaining 20% of CrEL contains 10% glycerol polyoxyethylene (Figure 1(c)), 7% free polyethyleneglycol (CrEL-PEG) (Figure 1(d)), and 3% nonreacted castor oil [3]. CrEL is used as a excipient in formulations for the solubilization of various hydrophobic drugs including anesthetics [4], antineoplastic agents [4, 5], immunosuppressive agents [6], analgesics [7, 8] vitamins [9], and new synthetic water insoluble compounds. Although CrEL is considered relatively nontoxic [4, 10], several reports suggest that drugs administered in CrEL induce serious complications like anaphylactoid hypersensitivity [9], axonal swelling, degeneration, and demyelination [5, 6]. Moreover, it has been proposed that CrEL plays a role in the etiology of peripheral neuropathy observed after intravenous paclitaxel [5] or cyclosporin A treatment and recent evidence supports the notion that CrEL causes nerve conduction [5, 11]. Pharmacokinetic disposition of drugs depends on CrEL pharmacokinetics [12]. There exists different analytical methods for the quantitative measurement of CrEL such as colorimetric [4, 13], chromatographic [14–16], electrophoresis [17], potentiometric [18–21], and pyrolysis mass spectrometry [22]. The colorimetric methods need very tedious preliminary procedures like cleavage and derivatisation of CrEL with an UV absorbing group. The chromatographic and electrophoresis methods need derivatisation and are time consuming. The potentiometric method suffers from limited sensitivity and stability. Pyrolysis mass spectrometry needs different mass spectrometry design, and throughput of analysis was a consideration. In the present work an attempt was made to develop and validate bioanalytical method for the quantitation of CrEL using LC-MS/MS and presented the plasma concentration profiles/PK parameters in male Sprague Dawley rats.

## 2. Experimental Section

### 2.1. Materials

Cremophor EL, dimethyl sulfoxide (DMSO), and polypropylene glycol (internal standard) were procured from sigma-Aldrich Co. (St. Louis, MO, USA). Acetonitrile, water, and tetrahydrofuran (THF) (HPLC grade) were procured from Merck Specialities Pvt Ltd. (Mumbai, India). Formic acid was procured from Merck specialities pvt ltd (Mumbai, India). Sprague Dawley rats were procured from Bioneeds Ltd. (Bangalore, India). Blood collection vacutainers (Lithium Heparin as anticoagulant) were sourced from BD (Franklin lakes, USA).

### 2.2. Preparation of Calibration Standards and Quality Control Samples

Master stock solution of polypropylene glycol (PPG) (1 mg/mL) (specific gravity: 1.01 g/mL) was prepared in DMSO. Master stock solution of CrEL (50 mg/mL) (specific gravity: 1.05 g/mL) was prepared in DMSO. Working standard solutions of CrEL-PEG were prepared by serial diluting from master stock with acetonitrile : DMSO : water (2 : 2 : 1). Working standard solutions were prepared at 25-fold higher concentration than plasma calibration standards and quality control samples. Plasma calibration standards were prepared at 1.00, 1.50, 5.00, 20.00, 70.00, 120.00, 160.00, 180.00, and 200.00 *μ*g/mL, and quality control samples were prepared at three levels, namely, 2.50 *μ*g/mL (LQC, low quality control), 125.00 (MQC, middle quality control) and 162.00 *μ*g/mL (HQC, higher quality control). Calibration standards and quality control samples of CrEL-PEG were prepared by spiking 2 *μ*L of the working standard solutions into 48 *μ*L of blank rat plasma. Purity details of CrEL were taken into consideration for preparation of calibration standards and quality control samples of CrEL-PEG [3]. The working solution for internal standard (10 *μ*g/mL) was prepared by diluting an aliquot of master stock solution with acetonitrile. Master stock solutions were stored at 4°C when not in use. 

### 2.3. Sample Preparation

A 50 *μ*L aliquot of plasma (blank control plasma, plasma samples from rats dosed with CrEL, blank plasma spiked with calibration standards, and QC samples) was pipetted into a 96-well polypropylene plate and extracted with 200 *μ*L of acetonitrile-containing internal standard. Samples were vortex mixed for 10 min at 1200 rpm and centrifuged at 3350 g for 10 min at 4°C. 50 *μ*L of supernatant was pipette transferred in to a fresh analysis plate and diluted with 450 *μ*L of water and 5 *μ*L aliquots were injected for LC-MS/MS analysis.

### 2.4. LC-MS/MS Analysis

All mass spectrometric estimations were performed on a sciex 3200 QTrap triple quadrupole instrument with Turboion Spray (AB Sciex, Toronto, Canada). The HPLC system consisted two of LC20AD UFLC pumps and a SIL HTC autosampler (Shimadzu, Kyoto, Japan). The stationary phase was XBridge C18 with 3.5 *μ*m particle diameter (Waters, Ireland). The column dimensions were 50 × 4.6 mm. The mobile phase flow rate was 1.0 mL/min. The mobile phase consisted of 0.1% formic acid in water as aqueous component and acetonitrile with 20% THF as organic modifier. A generic gradient LC method with a short run time of 3.5 min was developed for the analysis of CrEL-PEG in plasma samples. The column and autosampler were maintained at 40°C and 4°C, respectively. The Turboion Spray source was operated with typical settings as follows: ionization mode, positive; curtain gas, 20 psi; nebulizer gas (GS1), 50 psi; heater gas (GS2), 50 psi; ionspray voltage, 5500V; temperature, 550°C. The molecular ions of CrEL-PEG and PPG were formed using the declustering potentials of 120V and 80V, respectively. In MRM mode, the most abundant and informative molecular ions corresponding to PEG oligomers (hydrophilic component, CrEL-PEG) were selected at *m/z* 828, 872, 916 and 960 and fragmented to identical daughter ion at *m/z* and 89.10 at collision energy of 50 V with medium CAD gas setting. In MRM mode, the most abundant and informative molecular ions corresponding to glycerol polyoxyethylene ricinoleate (hydrophobic component) were selected at *m/z*, 844, 888, 932, and 976 and fragmented to identical daughter ion at *m/z* 307.10. Molecular ion (*m/z* 906.80) of PPG was fragmented to daughter ion with *m/z* 117.10 at collision energy of 45 V. Peak areas for all components were automatically integrated using Analyst software version 1.5.

Enhanced scan functions in QTRAP, namely, enhanced MS scan coupled with enhanced product ion (EMS + EPI scan), was used to study the fragmentation pattern of hydrophilic and lipophilic oligomers that were separated in retention on a chromatographic column. A 30 min LC gradient (time (min)/%B = 0.01/5, 2.00/5, 15.00/55, 20.00/95, 25.00/95, 25.01/5, and 30.00/5) was developed to separate the oligomers on a c18 column (150 × 2.0 mm, 3.5 *μ*m) at a flow rate of 0.50 mL/min. Mobile phase consists of 0.1% formic acid in water as aqueous component and acetonitrile with 20% THF as organic component. 

### 2.5. Plasma Stability Determination of CrEL

Master stock solution (50 mg/mL) of CrEL was prepared in DMSO. 10 *μ*L of the master stock was spiked in 490 *μ*L of plasma to obtain a final concentration of 1 mg/mL. Samples were incubated for 1 hr at 37°C in male sprague dawley rat plasma to determine the plasma stability of the excipient studied. Samples were incubated in Thermomixer (Eppendorf, Germany) at vortex speed of 600 rpm. Reaction was terminated at different time points (5, 10, 20, 40, and 60 min) by precipitating 50 *μ*L of incubation mixture with 200 *μ*L of acetonitrile-containing PPG as internal standard. 0 min control samples were prepared in heat inactivated plasma (heated at 70°C for 5 min) and 50 *μ*L of the sample was precipitated with 200 *μ*L of acetonitrile containing PPG as internal standard. Samples were vortex mixed for 10 min at 1200 rpm and centrifuged at 3350 g for 10 min. 50 *μ*L of supernatant was transferred into a fresh analysis plate and diluted with 450 *μ*L of methanol : water (1 : 1). 5 *μ*L aliquots were injected for LC-MS/MS analysis.

### 2.6. Method Validation

Three precision and accuracy batches, consisting of calibration standards (1.00, 1.50, 5.00, 20.00, 70.00, 120.00, 160.00, 180.00, and 200.00 *μ*g/mL) were analyzed on three different days to complete the method validation. In each batch, QC samples at 2.50, 125.00, and 162.00 *μ*g/mL were assayed in sets of six replicates to evaluate the intra- and interday precision and accuracy. The percentage deviation of the mean from true values, expressed as relative error (RE), and the coefficient of variation (CV) serve as the measure of accuracy and precision, respectively. The selectivity was evaluated by analyzing blank plasma samples obtained from different animals. Extraction efficiency of CrEL-PEG was determined by comparing peak areas of analyte spiked before extraction into the six different lots of plasma with those of the analyte postspiked into plasma extracts. Matrix effect was evaluated from matrix factor values. Matrix factor was calculated by dividing mean peak areas of analyte post spiked in to plasma extracts with those of analyte spiked in to neat solutions at three QC levels. To assess post-preparative stability, six replicates of QC samples at each of the low, mid, and high concentrations were processed and stored under autosampler conditions for 24 h before analysis. To assess bench top stability, six replicates of QC samples at each of the low, mid, and high concentrations were kept at room temperature for 8 h before analysis. Freeze thaw stability was assessed at three QC levels for three freeze thaw cycles. To assess long-term stability, six replicates of QC samples at each of the low, mid, and high concentrations were kept at −80°C for 90 days before analysis.

### 2.7. Application

Individual rats (male Sprague Dawley) were dosed at 0.26 g/kg intravenously (Bolus) through tail vein and 0.26 g/kg orally through oral gavage needle. Dosing volume administered was 5 mL/kg. The composition of dosing vehicle used for the study was ethanol/CrEL/water (10 : 5 : 85, %v/v) [23, 24]. Serial blood samples were collected into vacutainers containing lithium heparin (anticoagulant) at 0.08, 0.25, 0.50, 1, 2, 4, 8, and 24 h postdose [25] after intravenous administration and 0.25, 0.50, 1, 2, 4, 8 and 24 h post dose [25] after oral administration. At each time point 200 *μ*L of blood was collected into vacutainers. Blood samples were collected using retro orbital puncture method. Plasma was isolated by centrifugation at 14,850 g for 10 min and stored frozen at −80°C until assay. Pharmacokinetic parameters such as elimination rate constant (Kel), half-life (*T*_1/2_), extrapolated drug concentration (*C*_0_), AUC_0–last_, AUC_0–inf_, AUC_%Extrapolated_, volume of distribution (*V*_*d*_), clearance (Cl), *T*_max⁡_,  *C*_max⁡_, and MRT last and absolute bioavailability were calculated using phoenix winnonlin software (v6.3). Absolute bioavailability was calculated using AUC_0–inf_ values as AUC_%Extrapolated_ was less than 20%.

## 3. Results and Discussion

### 3.1. LC-MS/MS Analysis

**Figure 2:**
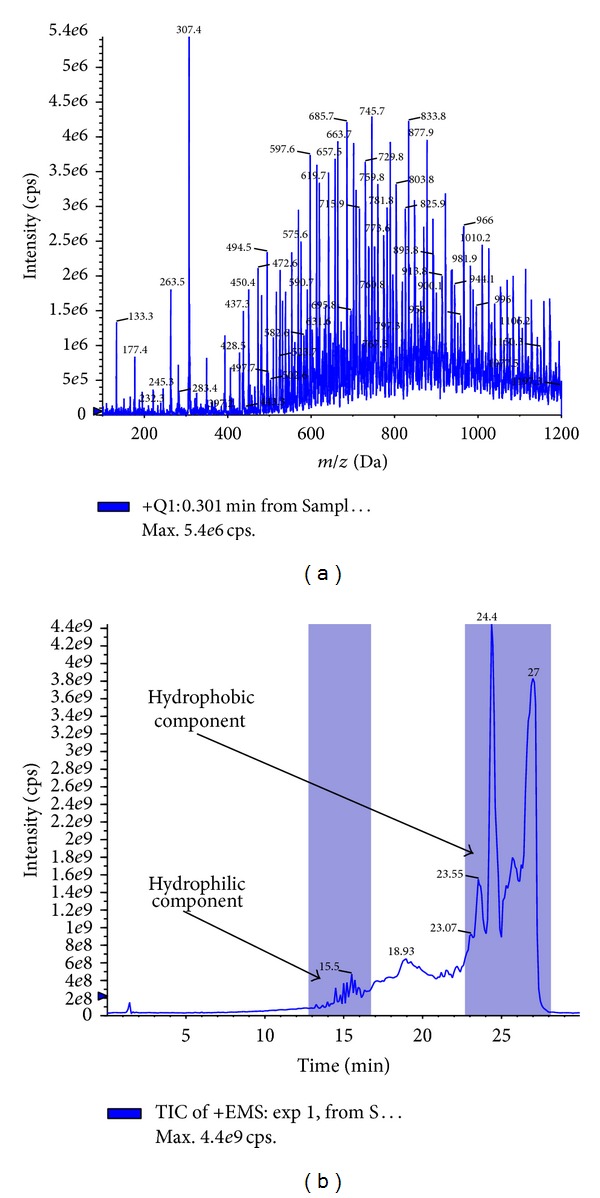
(a) Parent ion (full scan) scan of CrEL. (b) Chromatogram representing the difference in elution pattern of hydrophobic and hydrophilic oligomers of CrEL.

**Figure 3:**
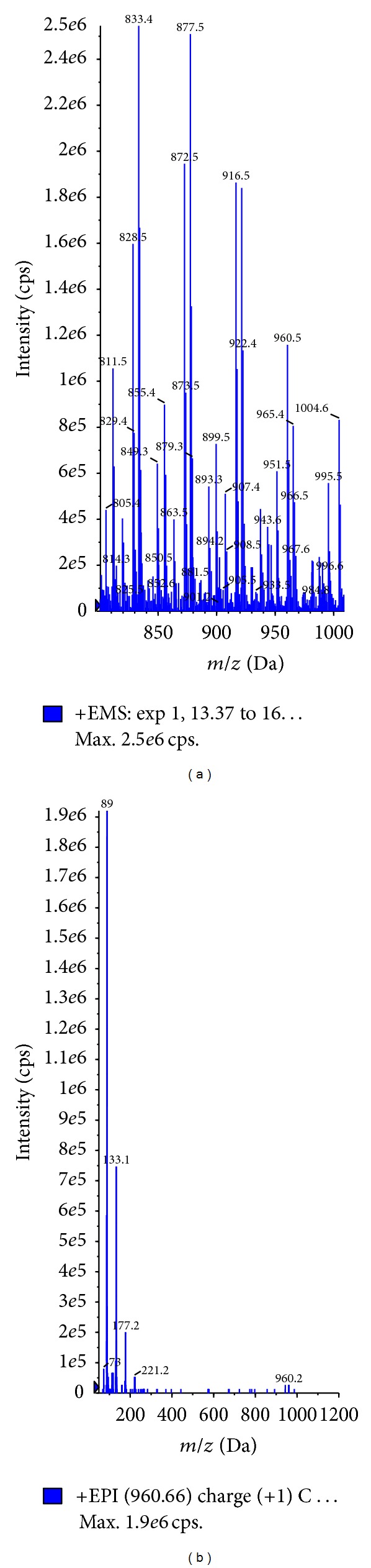
(a) Parent ion scan representing the molecular ions of PEG oligomers of CrEL. (b) Fragmentation pattern of selected oligomer with *m/z* 960.20.

**Figure 4:**
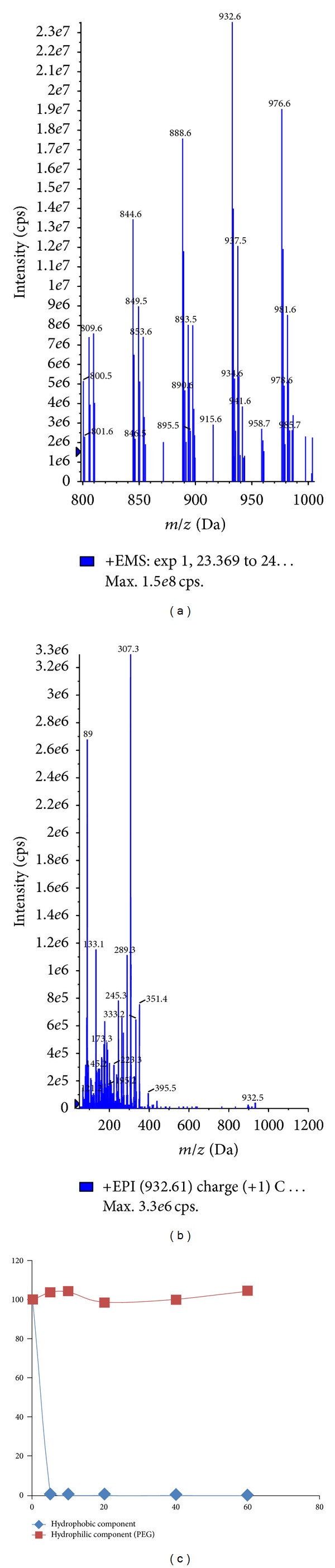
(a) Parent ion scan representing the molecular ions of hydrophobic oligomers (glycerol polyoxyethylene ricinoleate) of CrEL (b) Fragmentation pattern of selected oligomer with *m/z* 932.50. (c) Remaining percent versus time curve representing the plasma stability of hydrophilic and hydrophobic components of CrEL.

**Figure 5:**
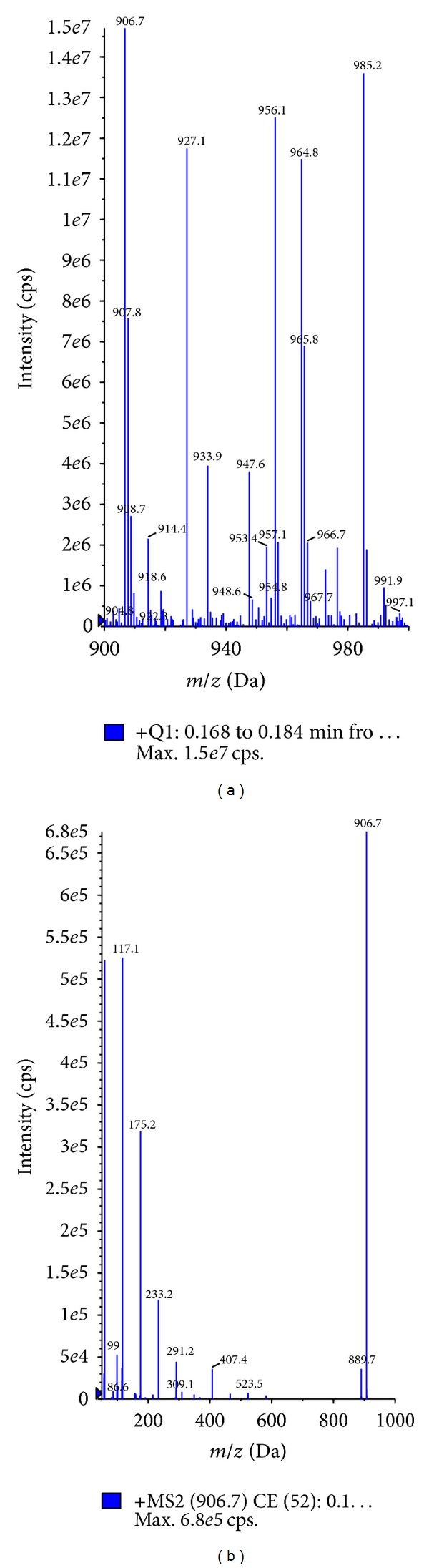
(a) Parent ion scan representing the molecular ions of PPG. (b) Fragmentation pattern for molecular ion of PPG with *m/z* 906.70.

The electrospray ionization of CrEL produced numerous molecular ions under positive ionization conditions (Figure 2(a)). It was difficult to pick the molecular ion of interest with too many molecular ions showing up in the spectra. Majority of the molecular ions did not produce any distinct fragment ions to analyze in MRM mode of analysis. So, in order to understand the molecular ions that correspond to hydrophilic and hydrophobic portions, tuning solution was injected through LC column in to mass spectrometer. Clear separation was achieved with the developed 30 min LC gradient (Figure 2(b)). Hydrophilic oligomers corresponding to glycerol polyoxyethylene did not generate any distinct daughter ions, whereas hydrophilic oligomers corresponding to PEG oligomers (Figure 3(a)) generated identical daughter ion with *m/z* 89.10 (Figure 3(b); fragmentation pattern of one oligomer *m/z* 960.20 was represented in the figure, other oligomers also shared similar fragmentation pattern). The molecular ions of PEG oligomers detected were ammonium adducts. For calculating the plasma concentrations of CrEL-PEG as a whole, analyte peak areas of the four oligomers were summed and calibration curve was built. Hydrophobic oligomers corresponding to glycerol polyoxyethylene ricinoleate with molecular ions at 844, 888, 932, and 976 (Figure 4(a)) generated distinct daughter ions with *m/z* 307.10 (Figure 4(b)). Determination of hydrophobic component, although with distinct fragment ions, was not taken up as it was found highly unstable in rat plasma (Figure 4(c)). Glycerol polyoxyethylene component (hydrophilic) was not taken up for analysis as there were no distinct fragment ions produced for any of the oligomers. The electrospray ionization of PPG produced abundant molecular ions at *m/z* 906.70 (Figure 5(a)) and generated an intense fragment at 117.10 amu (Figure 5(b)). LC-MS/MS method operated with the C18 column and a 3.5 min generic gradient LC method (Time (min)/%B = 0.01/5, 1.50/95, 2.50/95, 2.60/5, and 3.50/5) was developed for the analysis of CrEL-PEG in plasma. Various organic modifiers such as acetonitrile, methanol, and acetone were tested for achieving better peak shape and address the response saturation observed at higher calibration standards for CrEL-PEG. All organic modifiers tested resulted in poor linearity with response saturation at higher calibration standards. However, addition of THF to acetonitrile resulted in better linearity and response saturation was not seen. THF is the solvent of choice for the analysis of many polymers [26, 27], but, higher percentages of THF cannot be used practically in LC-MS/MS as it is highly inflammable solvent. So, final mobile phase conditions were optimized to 20% THF in acetonitrile. 

**Figure 6:**
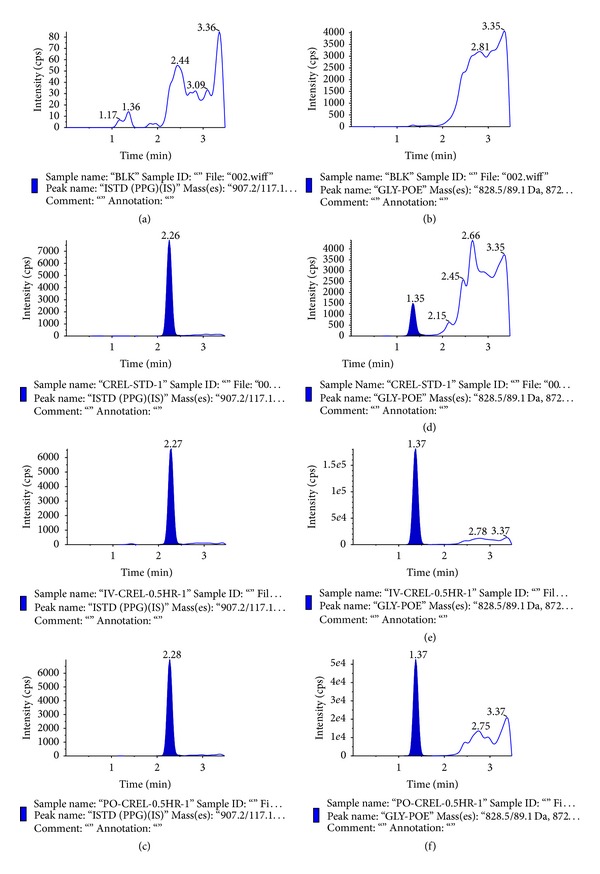
MRM LC-MS/MS chromatograms of (a) PPG in blank rat plasma; (b) CrEL-PEG in rat blank plasma; (c) PPG spiked at 10 *μ*g/mL concentration in rat plasma; (d) Rat plasma sample spiked with 1.00 *μ*g/mL of CrEL-PEG; (e) plasma sample obtained 0.50 hr after intravenous administration of CrEL to SD rats; (f) plasma sample obtained 0.50 hr after oral administration of CrEL to SD rats.

Because of the higher sensitivity of LC-MS/MS method compared to that of HPLC or colorimetric methods, lesser plasma sample volume (50 *μ*L) is sufficient to obtain an LLOQ of 1.00 *μ*g/mL. Colorimetric methods lacked detection sensitivity with an LLOQ of 500 *μ*g/mL. Even though the calibration range of 1 *μ*g/mL to 200 *μ*g/mL was higher for analysis on mass spectrometer, analysis of plasma samples revealed that the plasma concentrations of CrEL was around 1 mg/mL in the initial sampling points from intravenous route. Therefore, if these study samples have to fit in to the low ng/mL standard curve, very high dilution (100–1000-fold) is required, which requires more blank plasma for dilution, which practically is a limitation in drug discovery. So rather than developing a method with high sensitivity, here efforts were put to develop fit for purpose bioanalytical method, by selecting the mass spectrometer (3200 QTRAP) that is not highly sensitive, diluting the precipitated samples 10-fold after precipitation and injected less volume of sample (5 *μ*L). No interference at the retention times of PPG (2.26 min) (Figure 6(a)) and CrEL (1.35 min) (Figure 6(b)) was observed in any of the lots screened as shown in representative chromatogram of the extracted blank plasma sample, confirming the selectivity of the present method. Representative chromatogram of PPG at 10 *μ*g/mL spiked concentration was shown in Figure 6(c). Representative chromatogram of CrEL at LLOQ was shown in Figure 6(d). The LLOQ was set at 1.00 *μ*g/mL for CrEL-PEG using 50 *μ*L of rat plasma. The retention times of CrEL-PEG and PPG were reproducible throughout the experiment and no column deterioration was observed after analysis of plasma samples.

### 3.2. Plasma Stability Determination of CrEL

Hydrophobic component was found very fastly hydrolyzing (ester hydrolysis) in rat plasma with <1% remaining at 5 min. This could be due to esterases present at higher levels in rat plasma causing hydrolysis of hydrophobic component of CrEL. Similarly, hydrophilic component was found stable in 60 min incubation period. As hydrophobic component was found highly unstable in the rat plasma, analysis of the free PEG oligomers (hydrophilic component) was taken up to understand the pharmacokinetic disposition of CrEL. 

### 3.3. Method Validation

This method was validated to meet the acceptance criteria of industrial guidance for the bioanalytical method validation [28]. The nine-point calibration curve obtained for CrEL-PEG was linear over the concentration range of 1.00–200.00 *μ*g/mL with mean correlation coefficient ≥0.9972. The ratio of area response for CrEL-PEG and PPG (internal standard) was used for regression analysis. Of the two weighing models (1/*x*,  1/*x*∗*x*) used for curve fitting, linear regression analysis (*y* = *mx* + *c*) with a weighting factor of 1/(*x*∗*x*) gave the optimum accuracy of the corresponding calculated concentrations at each level (Table 1). The mean accuracy (RE%) and precision (CV%) observed for the calibration curve standards ranged from −6.22 to 5.33% and 0.41 to 7.77%, respectively.  Table 2 shows a summary of intra- and interday precision and accuracy data for QC samples containing CrEL-PEG. Both intra- and interassay CV values ranged from 0.69 to 9.21% at three QC levels. The intra- and interassay RE values for CrEL-PEG were −7.60 to 4.74% at three QC levels. These results indicate that the present method has an acceptable accuracy and precision. As shown in Table 3, the overall extraction efficiency of CrEL-PEG was 103.23%, which was consistent with a total CV% less than 5% at three QC concentration levels. Mean matrix factor values of 1.05 (Table 3) at three QC levels show that the developed method is totally free of matrix effects. Acceptable matrix factor range for qualifying the method to be free from matrix effects is 0.85–1.15. Protein precipitation has been successfully applied to the extraction of CrEL-PEG from rat plasma. Extracted QC samples were stable when stored at 4°C for 24 h (autosampler stability) prior to injection, with <4% difference from theoretical concentration (Table 3). Spiked QC samples were stable when stored at room temperature for 8 h (bench top stability) prior to injection, with <2% (Table 3) difference from theoretical concentration. Spiked QC samples were stable for three freeze thaw cycles (freeze thaw stability) with <5% difference from theoretical concentration. Long-term stability at −80°C was proved for a period of 90 days with <3% (Table 3) difference from theoretical concentration (Table 3). 

### 3.4. Application Study

**Figure 7:**
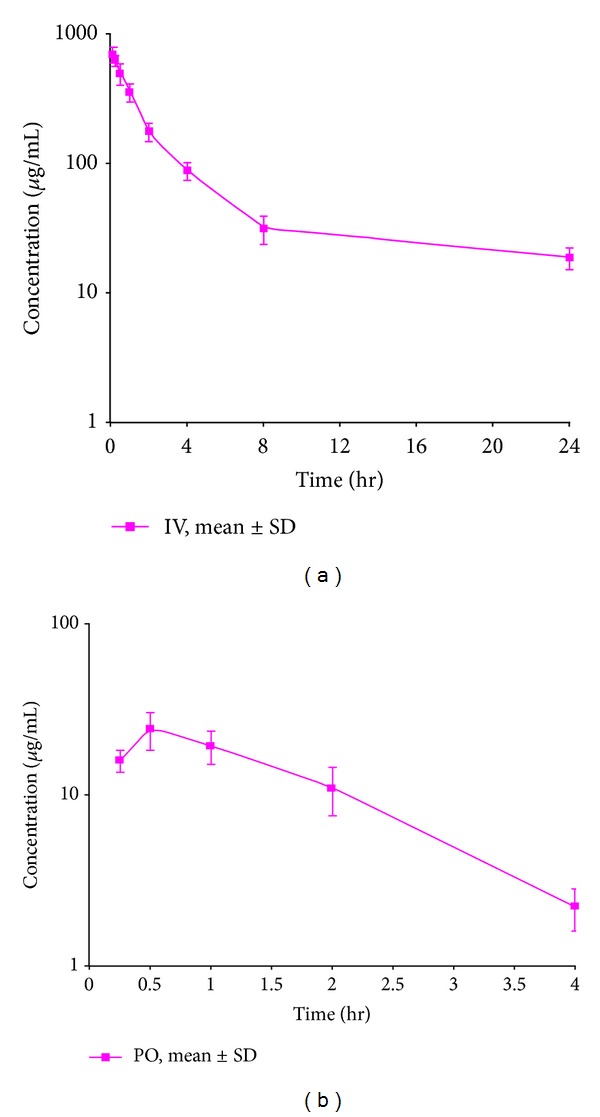
Mean Concentration time profile of CrEL (CEL-PEG) after (a) intravenous administration at 0.018 g/kg (PEG component) dose to SD rats (b) oral administration at 0.018 g/kg (PEG component) dose to SD rats.

This method has been successfully applied to the bioanalysis of rat plasma samples in absolute bioavailability study of CrEL. Representative chromatograms of CrEL-PEG from intravenous (0.50 hr), oral (0.50 hr) study samples were shown in Figures 6(e) and 6(f) respectively. The Intravenous and oral concentration/time profiles of CrEL-PEG is represented in Figures 7(a) and 7(b), respectively. As CrEL-PEG a clear absorption and elimination phase in oral route of administration and clear elimination phase in intravenous route of administration, measuring the excipient concentration levels along with NCEs helps to take a decision on the spiky profile of NCEs. Monitoring formulation excipient concentrations in PK study samples acts as quality control check for in vivo and bioanalytical processes. Intravenous and oral Pharmacokinetic parameters of CrEL-PEG were listed in Tables 4 and 5 respectively. The oral bioavailability of CrEL-PEG was measured as 2.80% which shows that the excipient had very poor absorption. Mean terminal half-life of CrEL-PEG after oral and intravenous administration was 1.26 hr and 7.65 hr respectively. Mean Tmax and Cmax after oral administration of CrEL-PEG to Sprague Dawley rats was 0.50 hr and 24.46 *μ*g/mL, respectively. Mean residence time of CrEL-PEG after intravenous and oral administration of CrEL to Sprague dawley rats was 5.25 and 1.62, respectively. Mean volume of distribution after intravenous administration (0.16 L/kg) was much lesser than total normalized body water 0.7 L/kg in rats. Mean clearance of CrEL-PEG after intravenous administration was much less (0.24 mL/min/kg body wt) than hepatic blood flow in rats (70 mL/min/kg body wt). 

## 4. Conclusion

A rapid, sensitive, and selective LC-MS/MS method for the determination of CrEL-PEG in rat plasma has been successfully developed and validated using protein precipitation extraction as sample preparation procedure. This assay method demonstrated acceptable sensitivity (LLOQ: 1.00 *μ*g/mL), precision, accuracy, selectivity, recovery, and stability. The validated method was successfully applied to assay rat plasma samples. The plasma concentration profiles/PK parameters of CrEL after intravenous and oral administration in male Sprague Dawley rats were established.

## Figures and Tables

**Table 1:** Calculated concentrations and statistical parameters of CrEL-PEG calibration standards prepared in rat plasma (*n* = 3).

Concentration (*µ*g/mL)				Statistical parameters			
Actual conc.	Calculated conc.			Mean	SD	CV%	Relative error (%)
	Set 1	Set 2	Set 3				
1.00	1.07	1.04	1.05	1.05	0.02	1.45	5.33
1.50	1.40	1.41	1.41	1.41	0.01	0.41	−6.22
5.00	4.56	5.15	4.95	4.89	0.30	6.14	−2.27
20.00	18.84	19.05	18.54	18.81	0.26	1.36	−5.95
70.00	70.19	67.98	68.39	68.85	1.18	1.71	−1.64
120.00	122.31	122.36	127.58	124.08	3.03	2.44	3.40
160.00	173.41	176.10	152.36	167.29	13.00	7.77	4.56
180.00	187.32	174.58	194.31	185.40	10.00	5.40	3.00
200.00	199.88	196.18	205.05	200.37	4.46	2.22	0.19

**Table 2:** Precision and accuracy of CrEL-PEG in quality control samples.

Type	Statistical parameter	Concentration (*µ*g/mL)		
		LQC (2.50)	MQC (125.00)	HQC (162.00)
Intraday Set 1 (*N* = 6)	Mean	2.47	130.93	164.61
	SD	0.13	10.94	15.16
	CV%	5.19	8.36	9.21
	Relative error (%)	−1.07	4.74	1.61

Intraday Set 2 (*N* = 6)	Mean	2.31	124.65	163.33
	SD	0.08	4.29	10.49
	CV%	3.60	3.45	6.42
	Relative error (%)	−7.60	−0.28	0.82

Intraday Set 3 (*N* = 6)	Mean	2.43	123.34	165.59
	SD	0.15	7.13	11.18
	CV%	6.08	5.78	6.75
	Relative error (%)	−2.80	−1.33	2.22

Interday (*N* = 3)	Mean	2.40	126.31	164.51
	SD	0.08	4.06	1.14
	CV%	3.52	3.21	0.69
	Relative error (%)	−3.82	1.04	1.55

**Table 3:** Summary of validation parameters for CrEL-PEG in rat plasma.

Validation parameter	Statistical parameter	Result
Extraction recovery	Mean	103.23
	SD	5.28
	CV%	5.11

Matrix factor (matrix effect)	Mean	1.05
	SD	0.03
	CV%	2.87

Autosampler stability	Mean	101.04
	SD	3.71
	CV%	3.68

Bench top stability	Mean	103.05
	SD	2.07
	CV%	2.00

Freeze thaw stability	Mean	100.73
	SD	4.55
	CV%	4.52

Long-term stability	Mean	104.65
	SD	2.89
	CV%	2.76

**Table 4:** Pharmacokinetic parameters of CrEL-PEG after intravenous administration at 0.018 g/kg dose (PEG Oligomers) in male Sprague Dawley rats.

Subject	Kel (1/hr)	*T*_1/2_ (hr)	*C*_0_ (*μ*g/mL)	AUClast (hr∗*μ*g/mL)	AUCINF_obs (hr∗*μ*g/mL)	AUC_%Extrap_obs (%)	Vz_obs (L/kg)	Cl_obs (mL/min/kg)	MRTlast (hr)
RAT-1	0.11	6.29	834.53	1714.61	1849.94	7.32	0.13	0.24	4.32
RAT-2	0.08	8.17	745.12	1566.77	1794.63	12.70	0.17	0.24	5.43
RAT-3	0.08	8.50	609.02	1620.79	1886.80	14.10	0.17	0.23	6.01
Mean	0.09	7.65	729.56	1634.06	1843.79	11.37	0.16	0.24	5.25
SD	0.02	1.19	113.56	74.81	46.39	3.58	0.02	0.01	0.86
CV%	17.03	15.60	15.56	4.58	2.52	31.49	15.73	2.53	16.33

**Table 5:** Pharmacokinetic parameters of CrEL-PEG after oral administration at 0.018 g/kg dose (PEG component) in male Sprague Dawley rats.

Subject	Kel (1/hr)	*T*_1/2_ (hr)	*T*_max⁡_ (hr)	*C*_max⁡_ (*μ*g/mL)	AUClast (hr∗*μ*g/mL)	AUCINF_obs (hr∗*μ*g/mL)	AUC_%Extrap_ obs (%)	Vz_F_obs (L/kg)	Cl_F_obs (mL/min/kg)	MRTlast (hr)	*F* (%)
RAT-1	0.61	1.14	0.50	18.42	45.01	49.82	9.67	0.86	8.78	1.62	2.62
RAT-2	0.85	0.82	0.50	30.50	46.85	49.07	4.52	0.63	8.92	1.31	2.99
RAT-3	0.38	1.84	0.50	24.45	45.29	48.21	6.04	1.44	9.08	1.93	2.79
Mean	0.61	1.26	0.50	24.46	45.72	49.03	6.74	0.98	8.92	1.62	2.80
SD	0.23	0.52	0.00	6.04	0.99	0.81	2.64	0.42	0.15	0.31	0.18
CV%	38.39	41.22	0.00	24.70	2.17	1.65	39.20	42.66	1.65	19.18	6.52
